# Nursing heroism in the 21^st ^Century'

**DOI:** 10.1186/1472-6955-10-4

**Published:** 2011-02-16

**Authors:** Philip Darbyshire

**Affiliations:** 1 Philip Darbyshire Consulting Ltd, PO Box 144, Highbury 5089, South Australia

## Abstract

**Background:**

The Vivian Bullwinkel Oration honours the life and work of an extraordinary nurse. Given her story and that of her World War II colleagues, the topic of nursing heroism in the 21^st ^century could not be more germane.

**Discussion:**

Is heroism a legitimate part of nursing, or are nurses simply 'just doing their job' even when facing extreme personal danger? In this paper I explore the place and relevance of heroism in contemporary nursing. I propose that nursing heroism deserves a broader appreciation and that within the term lie many hidden, 'unsung' or 'unrecorded' heroisms. I also challenge the critiques of heroism that would condemn it as part of a 'militarisation' of nursing. Finally, I argue that nursing needs to be more open in celebrating our heroes and the transformative power of nursing achievements.

**Summary:**

The language of heroism may sound quaint by 21^st ^Century standards but nursing heroism is alive and well in the best of our contemporary nursing ethos and practice.

## Background: Is heroism possible after Vivian Bullwinkel?

Any author addressing the topic of "Nursing Heroics: what it means in the 21st Century" faces a challenge and even more so when they take this on as the Vivian Bullwinkel Memorial Lecture.

They are perhaps doomed before they begin, for what modern day nursing heroism outside of a war zone is ever going to be able to withstand comparison with the experiences of Sister Bullwinkel and her colleagues - during the fall of Singapore, the sinking of the Vyner Brooke, the murderous machine gunning of Vivian and her 21 fellow nurses on Radji Beach and her years of internment in the death camps of Sumatra.[1-3]

Somehow, to mention any other forms of heroism in the same breath seems almost disrespectful, a feat of linguistic relativism designed to dilute or trivialize the power of the term. But please, bear with me as I explore the idea and the practices of nursing heroism in our current age.

## Discussion

### Today's nursing heroism

First, the more traditional concept of heroism as courage and providing service to others in the face of extreme personal danger is undoubtedly alive and well in nursing and in other human services. Firemen still enter burning buildings to save their occupants and nurses still join their health care colleagues in providing care to the hungry, the fearful, the injured and the dying in both natural disaster areas and man made conflict zones. Haitian nursing students and faculty from the Episcopal University of Haiti, were setting up first aid stations to help the victims of their city 30 minutes after that country's massive earthquake[4]. Military nurses and nurses from voluntary organisations such as Red Cross and Medicine Sans Frontiers are found in every war zone, every famine-blighted country, every dictatorial wasteland, every manifestation of 'hell on earth'. We fervently wish that the circumstances that draw them away from their own families and homes to these places did not exist, but they do, and thankfully, these nurses continue to respond.

### HIV/AIDS, SARS and nurse heroism

Consider also, nurses' responses to the fear and danger surrounding the emergence of infectious outbreaks such as HIV/AIDS in the 1980s and SARS in 2003. In the early 1980s when first reports were emerging of young gay men in the USA dying of seemingly systemic immune system failure, we could not have realised that this thing called 'GRID' was the start of the AIDS pandemic that has claimed the lives of more than 25 million people worldwide and has left approximately 33.4 million people living with HIV/AIDS[5].

During these times we saw the best and worst of nursing and health care. In an Oral History project: "The AIDS Epidemic in San Francisco: The Response of the Nursing Profession, 1981-1984[6,7]" we hear from nurses involved that some nursing and medical staff held the same fears and prejudices that were so widespread in the broader community. They would refuse to care for the AIDS patients and stigmatise them along with the other so-called "4H patients - Homosexuals, Haitians, Hemophiliacs and Heroin users"[8].

Helen Miramontes, who later became one of the world's leading nurse advocates, specialists and educators in HIV/AIDS was then a clinician. She said that: *"There was a lot of fear among health care providers about contagion, but there was also significant prejudice and discriminatory behavior because the new disease was identified in a population that was stigmatized by the larger society. Identification of the disease in people of color, especially African Americans and injection drug users, only exacerbated the biases, prejudices, and discriminatory behavior. Many nurses demonstrated the same attitudes, beliefs, and behaviors seen in the larger society. I was a critical care nurse working in an intensive care unit (ICU) in a large teaching facility. In the early years of the epidemic, it was not unusual to have two to three patients with Pneumocystis carinii pneumonia on ventilators in the ICU at any one time. Because some nurses avoided taking care of these patients, several of us volunteered to care for them on a regular basis. There were frequent breaches in confidentiality, not only among nurses but also among other health care workers"*[7].

Gary Carr, who was a Nurse Practitioner at the AIDS Clinic at San Francisco General Hospital, described the perverse ambivalence of a wider community that lauds and praises nurses for their 'heroic efforts' in the face of such public health crises. Gary says: *"I have no memories of being afraid or being brave. I just wasn't afraid. I just said to myself, this is what I want to do. This is important. The community needs this, and it's what I want to do. I remember there were people who stopped speaking to me. My mother for years didn't tell anybody what I did. My relatives for years thought I still worked in the trauma unit"*[6].

When, two decades later, SARS emerged as a potentially lethal viral infection, nurses and health care staff again faced considerable dangers as they strove to treat patients and protect their communities. Dr Dessmon Tai, who led the Singapore efforts against SARS, wrote that: *"No other disease had such a phenomenal impact on healthcare workers."*[9]

But it seems that something in our professional ethos had changed over these two decades. There had been a rediscovery or reaffirmation of our professional ethic and mission as nurses, doctors and health professionals. In Hanoi, during the initial outbreak, rather than look for an 'opt-out' clause in their professional codes, doctors and nurses locked themselves into the hospital in isolation rather than risk spreading the disease[10].

As Emmanuel notes: *"More than half of the first 60 reported cases of SARS involved health care workers who had come into contact with SARS patients. Indeed, apart from the very first case, all of the people who died in Vietnam were doctors and nurses. Nearly a quarter of all patients with SARS in Hong Kong were health care workers. In Canada, of the 141 probable cases of SARS diagnosed between 23 February and 14 May 2003, 92 (65%) involved health care workers. Despite deadly peril, physicians and nurses tirelessly cared for patients with SARS"*. [8]

However, the social stigma that surrounded HIV/AIDS twenty years earlier and the associated ambivalence of the community towards health care professionals was not so easily repressed. In Toronto, Canada, Hall and colleagues reported that: *"children of nurses were barred from school trips and families were shunned by their neighbours. Other incidents included husbands of nurses being sent home from work, children of nurses shunned at school, nurses refused rides by taxi drivers, and single-parent nurses unable to get babysitters"*.[11]

Similarly, in Singapore, nursing staff were reportedly shunned in public spaces, forbidden to use the lifts in their apartments, found that buses and taxis would not stop for them and as one review reported, *"At any packed food court, there would always be a seat for a Tan Tock Seng Hospital nurse. Queue lines would quickly shorten when a nurse joined that queue"*.[10]

These personal travails were compounded by what many saw as the unavoidable violence done to some of the best traditions of the nurse-patient relationship by the nature of the SARS virus and its containment. Nurses working with SARS patients were often isolated from collegial support, asked to eat meals alone and prohibited from attending meetings. Rigorous isolation and anti-infection procedures saw nurses, effectively in spacesuits, caring for patients in enforced cubicle isolation. If this was a terrible way to be ill, it was an even more solitary and disconnected way to die.

Yet despite these dangers and demands, nurses and our health care colleagues exemplified the best of who we are and what we do. They worked in a cauldron of contagion, initially unaware of what they were fighting, how the infection spread, how it killed or how it could be treated. As one French doctor commented, *"We were not playing with fire. It was playing with us"*[9]. Was this heroism and heroic actions on their part, or were they 'just doing their job'? If we accept that heroism is "providing service to others in the face of extreme personal danger", then I have no qualms in considering these nurses as exemplars of 21^st ^Century Nursing heroism.

### Heroism and 'militarism': What's in a word?

Let me sidetrack slightly at this point to address a concern about the very legitimacy and appropriateness of the term heroism in nursing. For some critics, the very mention of 'wars against disease', 'defeating illness', 'the battle against SARS', or 'nursing heroism', is tainted. The concern is related to the militaristic or combative nature of such language and how this might shape not only our understandings of illness and disease but also our understanding of nursing itself and indeed the content and foci of our education, research and services.

The concerns are not new, having been articulated most forcefully and influentially by the late American essayist, critic and author, Susan Sontag in both her books: 'Illness as a Metaphor' and 'AIDS and its Metaphors'[12]. Sontag was deeply critical of the metaphorical language that scaffolds our understandings of, in these cases, Cancer and AIDS. She rejects metaphors of battle, war, magic bullets, invasion, surrender, attack etc and with AIDS is even more scathing of its damning metaphorical encumbrances around divine retribution, plague, sexual contagion and societal decay.

*"The body is not a battlefield" *says Sontag[12], but when faced with illness or injury, I suspect that it may become one, if for no other reason that nurses, people and patients need something to fight against. People will often accept the most devastating of diagnoses with almost a gratitude that seems completely misplaced, until they explain to us that the previous uncertainty or not knowing had been far, far worse.

Author and doctor, Peter Goldsworthy depicts this phenomenon beautifully in his celebrated novella, 'Jesus Wants Me for a Sunbeam' which describes how a family reacts when their young daughter develops leukaemia:

*"For Rick and Linda there was also, at the end of that terrible week of waiting and worry, an odd feeling of relief that it had happened to them, and theirs. Anything was better than uncertainty; the waiting had been intolerable, the fear of the unmentionable had almost come to be a desire for the unmentionable; its certainty, its mention, was at least a resolution. To finally hear the word (leukaemia) spoken aloud provided a focus for worry, a definite enemy that they could now face, and fight, together, as a family." *[13]

Before leaving the subject of language, let me touch on a recently articulated concern about the language and discourses of militarism and how these may influence Nursing.

In a new paper this year on the Politics of Nursing Knowledge, Perron and her colleagues[14] criticise what they call the 'militarization of nursing', claiming that: *"Many accounts provide angelic portrayals of nurses faced with the devastating effects of war. Such nurses are described as loyal, beautiful, peaceful, healing, comforting, reliable, devoted, and courageous in the face of hardship. These romantic descriptions stand in sharp contrast to the organized killing and destruction of war-making."*[14]

I would argue that what Perron and her colleagues have almost studiously overlooked are that these allegedly 'romantic descriptions' also stand in sharp contrast to any respectable historical account of the experiences of military nurses[1]. In such histories, and in particular the growing collection of oral histories of nurses' wartime and conflict zone experiences[3,15-19], we will find not saccharine, sentimentalized, spin-doctoring but vivid recollections and narratives from nurses, who, in addition to thankfully being 'loyal, comforting, devoted, healing, reliable and courageous', are also intelligent, skillful, determined, demanding, creative and resourceful.

Heather Höpfl, a Professor of Management, takes a quite different, and I believe more coherent and enabling view of women's heroism than Perron and her colleagues. She argues that: *"The heroines of history are not impotent women. Quite the contrary, they are women who refuse to be put in their place. The stories of heroines offer a picture of women who were far from meek and far from conciliated into male reality definitions. They were real women not mythological constructions. They shared values rather than common backgrounds and they are, to use an unfashionable term 'indomitable'. (...) The stories of the heroines of 50 years ago and more are stories of recusancy. They are stories of opposition. They are in almost every case stories of gender politics. We are deceived when we are told they are stories of female oppression. (...) These women faced enormous obstacles but squared themselves off against them and responded with integrity and courage." *[20]

### Of other 'quiet' and 'unrecorded' heroisms

Let me now speak of other heroisms, for I believe heroism in nursing to be a broad church, a continuum of courage rather than a zero-sum game. The nurse exhibiting what I might call, after Dickens' character, Sydney Carton, a "quiet heroism", or what Patricia Benner calls "unrecorded heroism"[21], continues to be part of the fabric of health care today.

Nursing may not be among the first occupations that springs to mind when the word 'danger' is mentioned but a recent Forbes magazine feature in the USA did identify nurses and nurses' aides as one of the top two groups experiencing among the highest rates of injury at work, albeit usually from lifting and handling incidents[22]. In addition, Hall and colleagues in the US reported that: *"Nursing assistants working in long-term care facilities have the highest incidence of workplace violence of any American worker"*.[23]

Nurses in our Emergency Departments experience perhaps even more severe episodes of violence. In Scotland, we used to joke that any particularly rough place was: "Like casualty on a Friday night", but it is no joke now. In a recent Australian survey from WA, Rose Chapman and colleagues found that: *"The majority (92%) of nurses said they had been verbally abused, 69% had been physically threatened and 52% had been physically assaulted". "Only 16% of the nurses completed an official incident report. Reasons for not reporting included the view that Work Place Violence is just part of the job, and the perception that management would not be responsive"*.[24]

Would Emergency Department nurses see themselves as 'heroes'? Almost certainly not, as they seem to have internalized a workplace culture where abuse and violence is not an aberration but simply something that "comes with the job"[25] and where reporting such abuse is scarcely worth the trouble. Perhaps if we return to the definition of heroism as 'providing service in the face of extreme personal danger', then our Emergency Department nurses should allow themselves to feel, at least somewhat heroic.

When we define heroism as providing service in the face of personal danger, that danger is not always the danger of illness, injury or death. Sometimes, that danger emanates from inside the very organisations that we serve and what is under threat is not nurses' bodily integrity but their professional and personal integrity. I refer of course to the phenomena of whistleblowing in nursing and health care.

Nursing's official pronouncements, policy directives, mission and vision statements, strategic plans, nursing philosophies and the like invariably proclaim our commitment to the highest standards (indeed to excellence), to patient safety, to quality care, to collegiality, to mutual respect, to 'patient-centredness', to patient advocacy and more. But for many nurses, there is not merely a gap between these aspirations and clinical reality, there is a Katherine Gorge.

Debra Jackson and her research team in Sydney have done some of the best Australian research in this area and it does not make for happy reading. As Debra notes: *"Currently, whistleblowing represents a professional dilemma and a personal disaster."*[26] This observation does not only apply to nursing. Medical whistleblowers fare equally badly at the hands of their own profession[27]. Indeed in all of the whistleblowing literature, it is difficult, if not impossible to find even one person whose principled actions have NOT resulted in huge personal and professional costs.

And yet we know that nurses are not only vital because of our numbers but because we are patient care, safety and quality where the rubber hits the runway. Nursing is not just some inert 'silo', needing to be dismantled. If a hospital or health authority does not understand the difference between nursing as a Silo and nursing as a Sentinel, they are in more trouble than they can possibly imagine. We are the world's best adverse patient experience early warning detection system. We are like the canaries down the mine or the frogs in the ecosystem. If nurses are metaphorically not singing or not croaking, if they are falling off the perch or disappearing from the ponds, then 'Huston - we have a problem"!

This problem is a phenomena that blighted not only Bundaberg Hospital[28], but other hospitals and health organisations across the world. This virulent, vocational virus - I'll call it: **MRSA **('Management Resistance to Staff Alerts'), has been implicated in almost every hospital 'scandal' and health system failure inquiry in recent years. This strain of 'MRSA' seems endemic in health care systems and is as dangerous to staff and patients as any hospital acquired infection. In a nutshell, health organisations and their leaders are not only failing to listen to their front line nursing staff, but in the worst cases, they are actively and forcefully trying to silence them.

At Bundaberg Hospital, what stopped Dr Jayant Patel was not a new computer system, it was not an updated reporting mechanism, it was not visits from external regulators, it was not another reorganization, it was not a new management theory. It was the bulldog advocacy and persistence of ICU Nurse Toni Hoffmann in the face of managerial inactivity and intimidation[28]. Toni was recognised for her role with an Australian Local Hero Award in 2006 and an Order of Australia in 2007. Is Toni Hoffmann a 21^st ^Century nurse hero? Without a shadow of doubt.

### Whatever happened to the heroes?

What of the other contemporary meaning of heroism and heroes? Who are our nursing heroes? Who are the nursing giants upon whose shoulders we have stood and who continue to inspire us today? Who are the nursing heroes that we tell our students about so that they can understand the best of Nursing's history? Who are they to emulate if they want to be the best nurse possible?

Our hardwired and often confused sense of egalitarianism makes us uncomfortable in even talking in such a way. When I ask nurses to tell me something wonderful that they did recently that made a real difference to a patient, client or family, their embarrassment and discomfort is almost immediately palpable. *'I didn't really do anything', 'it was the team', 'I'm just a nurse', 'I don't like to 'big note' (brag about) myself', 'All I did was..., 'I don't know what you mean'...*.

Please, let us be more open and unabashed in celebrating our nursing heroes. If these were sportsmen or sportswomen, if they were musicians or actors, if they were business people: we would be lauding their finest performances, dissecting their latest work with relish and handing out awards and trophies by the cabinetful.

I thought of my heroes for this paper, the nurses who have inspired me and who continue to do so. So let me celebrate, in no particular order of merit:

#### Virginia Henderson

Possibly the most lucid and coherent writer on nursing ever. I read her little 50 or 60 page book, 'Basic Principles of Nursing Care' as a young student nurse and could recite her definition of nursing, even at parties after a few drinks, as if it were a catechism. It is no exaggeration to say that I understood nursing in a completely different light after Virginia Henderson.

#### Patricia Benner

As a new PhD student grappling with philosophy, phenomenology, qualitative research approaches and new understandings of practice, reading 'The Primacy of Caring', followed by 'Novice to Expert' was to have the scales fall from one's eyes and to see and understand the world anew. The quality of Patricia Benner's thinking and scholarship is matched only by her graciousness and generosity of spirit.

#### Margaret Alexander

Margaret was Dean and Head of School when I took up my first post-PhD Lecturing position at Glasgow Caledonian University and was simply the Dean from heaven. Her standards and expectations were exhilaratingly exacting and her work rate would have shamed a Chilean mine rescuer. Her enthusiasm for nursing, for education, for research and for innovation and creativity was boundless. Her guiding philosophy seemed to be: 'The answer's yes, now what's the question'. How could you fail to thrive and develop as a new Faculty member under such inspiring and utterly humane leadership?

#### Linda Aiken

Linda Aiken is undoubtedly the doyenne of contemporary nursing research and one of the most powerful voices in nursing, in that when Linda Aiken's research speaks, the world of healthcare listens. And so it should. Her exemplary international research programme at the Center for Health Outcomes and Policy Research at the University of Pennsylvania demonstrates clearly that nursing is not simply one of many factors involved in improving health outcomes and patient safety, it is THE KEY FACTOR. Get nursing right and you improve safety, quality and patient care. No ifs, no buts.

#### Dodie Bryce

Dodie Bryce was an enrolled nurse at the intellectual disability hospital where I trained as a new nurse in the 1970s. These institutions could be grim places but Dodie Bryce's calm, humane, compassion and exemplary human caring skills made her truly, a light in the darkness. She was not just a great nurse but a presence. Older and wiser, I now understand the difference.

#### Maggie Watson

Was a ward sister at the Sick Children's Hospital in Edinburgh when I trained in pediatrics. I was simply in awe of her. She managed, as the sole charge nurse, a 30 bed surgical ward, the attached neonatal unit of around 6-10 cots and an attached 2 bed cardiothoracic surgery unit. She knew every child, everything about their condition and its care and seemingly all of their families as well. She was unflappable in any crisis and utterly respected and listened to by every doctor and health professional. That she managed to take time and trouble to help students on the ward like myself made her even more remarkable.

#### Sara Fleming

Is South Australia' first Nurse Practitioner and heads our Pediatric Palliative Care service. She was also my first clinical research collaborator when I took up my Joint Chair position at Women's & Children's Hospital. Sara is a human dynamo, possessed with vision, drive and absolute determination. Give a hospital half a dozen Saras in clinical leadership positions and they could rule the world.

#### Debra Jackson

Debra was my PhD student and the PhD student of every supervisor's dreams. Debra has a fierce intelligence matched only by her unstinting work ethic. When you combine these with a heart and personality that draws the best out of everyone around her, you can see why she now heads what is easily one of Australia's best research centers.

Who are the heroes on your list and why?

### The business of Nursing?

We are told constantly that health care is a business and that nursing should follow more business-like principles. As a health service or hospital is indeed a multi-million dollar organisation that needs to be well managed, there are no arguments from me on that score.

If we are in business however, then let us be absolutely clear about the nature of our business as nurses. We are in the transformation business and the 'making a difference' business. Nurses don't just make the tea and coffee they make decisions. We need to appreciate the importance of processes and structures, but more importantly, we need a laser focus and a near-reverence for tangible and valued outcomes that improve patients' experiences.

We are in the transformation business. As a clinician, you are not in the injections business or the dressing-changing business or the putting up IVs business or the bathing business.

Instead, as Kerfoot notes, we transform [29]

"We transform a frightened 4-year-old girl in the emergency room into a little person who now feels she has some measure of control and can stop crying. We transform a 50-year-old father with out-of-control diabetes into a person who has the confidence to manage his condition. And we transform the frightening and painful experience of childbirth into a beautiful memory of ecstasy for a family that has created a new person. When life ends, we transform those final moments of life into sacred, beautiful transitions of passage for families to complete the circle of life."

As a nurse educator, you are not in the business of 'lecturing', 'marking', 'supporting students', or 'writing curricula'. You are in the transformation business.[30]

We transform students into safe, skilled and self-confident practitioners.

We transform apathy and cynicism into enthusiasm and robust idealism.

We transform clinical, interpersonal and ethical problems from potential career-ending setbacks, into opportunities for deep learning and personal and professional mastery.

We transform patient and client experiences from everyday anecdote into the bedrock of clinical judgement and service quality.

We foster and build confidence and self-belief where this been eroded, damaged or has never developed while also challenging an equally dangerous overconfidence, arrogance or narcissism.

As a nurse researcher, you are not in the business of interviewing, administering surveys or managing data.

We transform the glib stereotype of the 'ivory-tower' academic by our meaningful, productive and mutually advantageous collaborations with clinical colleagues and service areas.

We challenge the prejudice that academics and their research has little relevance or use in the 'real world' of health policy and politics by our focus on knowledge translation, transfer and research impact and by the demonstrable profile and presence that our work has in numerous key areas of health policy and politics.

We are in the transformation business and the 'making a difference' business.

All over the world, nurses are making rhetorical notions of 'The Patient Experience', Quality & Safety and Improved Outcomes very, very real:

Somewhere a nurse is helping a struggling and despairing new mum to learn all of the messages and nuances that her new baby is signalling,

Somewhere a nurse is bearing witness to another mother's dying, and comforting her during her last moments on this earth,

Somewhere a nurse is inserting a child's IV and helping them and their family begin their journey into the world of chemotherapy,

Somewhere a nurse is listening to an Alzheimer's patient tell a story and trying to help them piece together who they really are,

Somewhere a nurse is helping a new student learn from a patient encounter and is passing on the wisdom of our art,

Somewhere a nurse is turning a hunch or a problem into a question that will eventually be researched and provide new knowledge and understanding,

Somewhere a nurse is managing a service with the passion and enthusiasm that enables her staff to thrive and to appreciate why they wanted to become nurses in the first place,

And somewhere a nurse is working in a war zone, helping service personnel and villagers alike.

These 'quiet' or 'unrecorded' heroisms surely deserve our acknowledgement and appreciation.

## Conclusion

At the end of her classic novel 'Middlemarch'[31], George Eliot writes an epitaph for her heroine Dorothea:

"But we insignificant people with our daily words and acts are preparing the lives of many Dorotheas...Her finely-touched spirit had still its fine issues, though they were not widely visible. Her full nature, like that river of which Cyrus broke the strength, spent itself in channels which had no great name on the earth. But the effect of her being on those around her was incalculably diffusive: for the growing good of the world is partly dependent on unhistoric acts; and that things are not so ill with you and me as they might have been, is half owing to the number who lived faithfully a hidden life, and rest in unvisited tombs."

So too, the health, wellbeing, safety and experiences of patients, clients and families are dependent upon the often invisible and overlooked caring practices of nurses. Today in the 21^st ^Century, they are worthy of sharing the term 'heroism' and I like to think that Sister Vivian Bullwinkel would agree.

## Competing interests

The author declares that they have no competing interests.

## Authors' contributions

PD is responsible for all aspects of this paper.

## Author's information

This paper is based on the 9^th ^Vivian Bullwinkel Lecture, given in Melbourne, Australia on 17^th ^November 2010. This annual event is hosted by La Trobe University, Monash University, the Royal College of Nursing Australia and the Nurses' Memorial Centre in memory of an outstanding nurse.

Author's position: Director, Philip Darbyshire Consulting.

Adjunct Professor, University of Western Sydney.

Honorary Visiting Professor, Swansea University.

Visiting Professor, Bournemouth University.

## Pre-publication history

The pre-publication history for this paper can be accessed here:

http://www.biomedcentral.com/1472-6955/10/4/prepub
